# Venous Plexus of Batson and Its Important Role in the Development of Cryptococcal Meningitis

**DOI:** 10.7759/cureus.114616

**Published:** 2026-08-16

**Authors:** Yuki Hironaka, Shoji Oura, Arito Kaji

**Affiliations:** 1 Department of Emergency Medicine, Kishiwada Tokushukai Hospital, Kishiwada, JPN; 2 Department of Surgery, Kishiwada Tokushukai Hospital, Kishiwada, JPN

**Keywords:** atypical meningitis symptoms, batson’s venous plexus, cryptococcal meningitis, cryptococcus neoformans, pigeon droppings

## Abstract

Cryptococcosis is an infectious disease caused by *Cryptococcus neoformans* and is most often found in the lungs, followed by the meninges. However, little is known about the mechanism by which cryptococcal meningitis develops without pulmonary cryptococcosis. A 78-year-old woman visited our hospital due to decreased appetite, difficulty moving, and fever. She had right-sided hemiparesis due to an old cerebral infarction, headache, no nuchal rigidity, a National Institutes of Health Stroke Scale of 4, and no pulmonary disorders on computed tomography. On the fourth day after hospitalization for symptom palliation, venous blood culture revealed *C. neoformans* infection. Given the high suspicion of cryptococcal meningitis, a lumbar puncture was performed and confirmed the presence of *C. neoformans* in the cerebrospinal fluid. The patient, therefore, began to receive amphotericin B (3.75 mg/kg) and 5-fluorocytosine (25 mg/kg) four times daily, leading to slow but definite improvement of the cryptococcal meningitis. Based on the clinical symptoms and outcomes of this case, we hypothesize that Batson's venous plexus may be involved in the development of cryptococcal meningitis without pulmonary cryptococcosis. Emergency physicians should note that meningitis resulting from this mechanism often does not present with typical meningitis symptoms.

## Introduction

Cryptococcosis is an infectious disease caused mainly by *Cryptococcus neoformans*, a fungus commonly associated with pigeon droppings [1]. The most common form of cryptococcal infection is pulmonary cryptococcosis, a well-known infection through the respiratory tract, followed by cryptococcal meningitis [2,3]. While some patients develop infections in both the lungs and the meninges, a significant number of patients develop cryptococcal meningitis without pulmonary cryptococcosis.

It is well known that cryptococcal infection frequently develops in patients with compromised immunity, such as those with acquired immunodeficiency syndrome [4]. Cryptococcal meningitis, however, requires hematogenous transmission of *C. neoformans* to the meninges whether the patient is immunocompromised or not. No studies, unfortunately, have reported how *C. neoformans* in the oral cavity, the most common site of entry of the fungus into the body, infects the meninges in patients with cryptococcal meningitis without pulmonary cryptococcosis to date.

We experienced a case of cryptococcal meningitis without pulmonary cryptococcosis, had difficulty in making the diagnosis due mainly to the lack of typical meningitis symptoms, and report on the possible pathogenetic mechanisms of such a specific disorder.

## Case presentation

**Figure 1:**
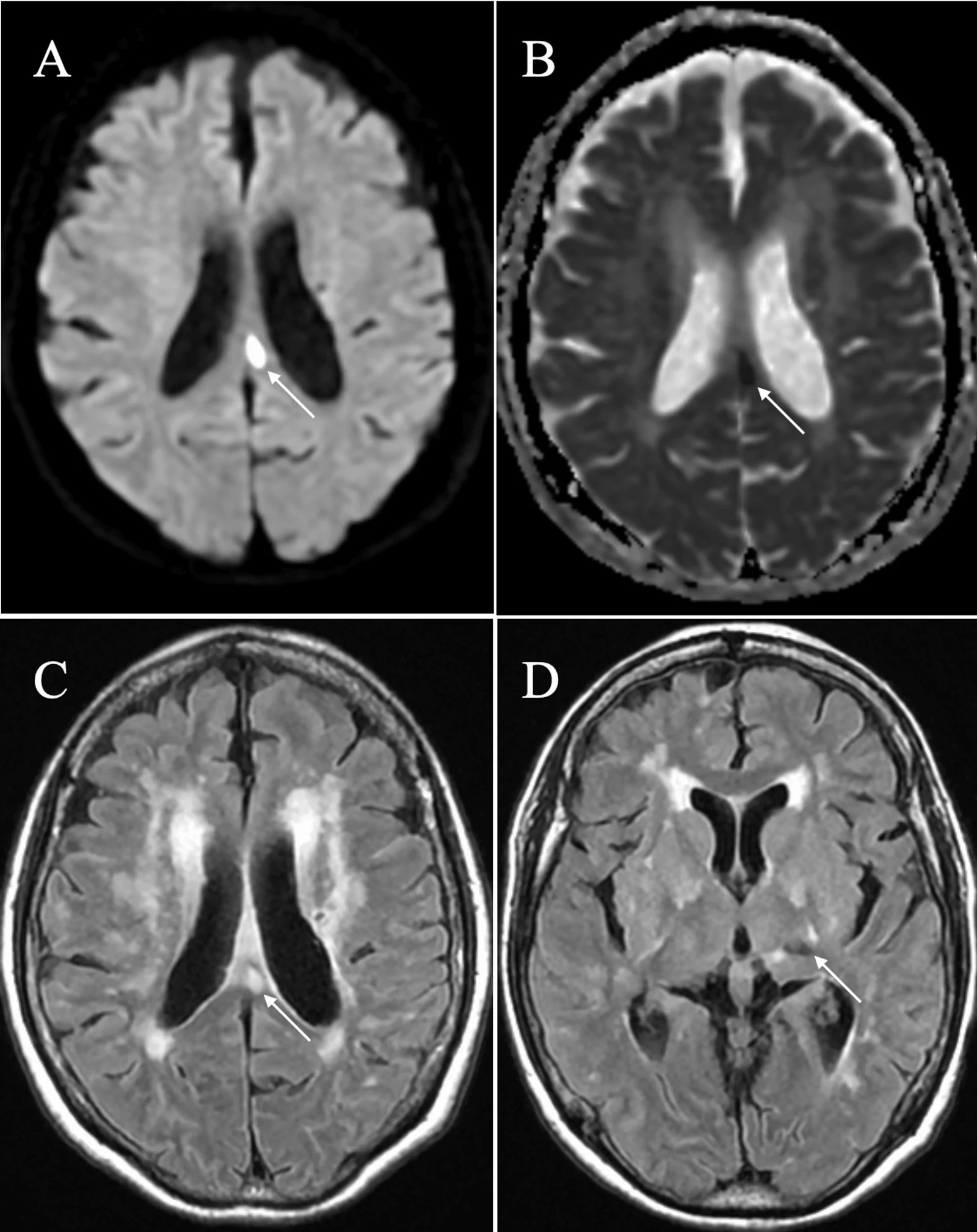
Brain MRI findings MRI showed a lesion in the splenium of the corpus callosum, showing high signals on diffusion-weighted images (A, arrow), low signals on apparent diffusion coefficient images (B, arrow), and high signals on fluid-attenuated inversion recovery images (C, arrow). FLAIR also showed a lesion in the left thalamus suggestive of an old cerebral infarction (D, arrows) MRI: magnetic resonance imaging; FLAIR: fluid-attenuated inversion recovery

A 78-year-old woman visited our hospital due to decreased appetite, difficulty moving, and fever persisting for several days. The patient was somewhat agitated and had difficulty communicating at the time of her first visit. She had right-sided hemiparesis due to an old cerebral infarction, headache, no nuchal rigidity, and a National Institutes of Health Stroke Scale of 4 [5]. Brain magnetic resonance imaging showed a lesion in the splenium of the corpus callosum showing high signals on diffusion-weighted images, low signals on apparent diffusion coefficient images, and high signals on fluid-attenuated inversion recovery images, as well as an old cerebral infarction in the left thalamus (Figure 1).

**Figure 2:**
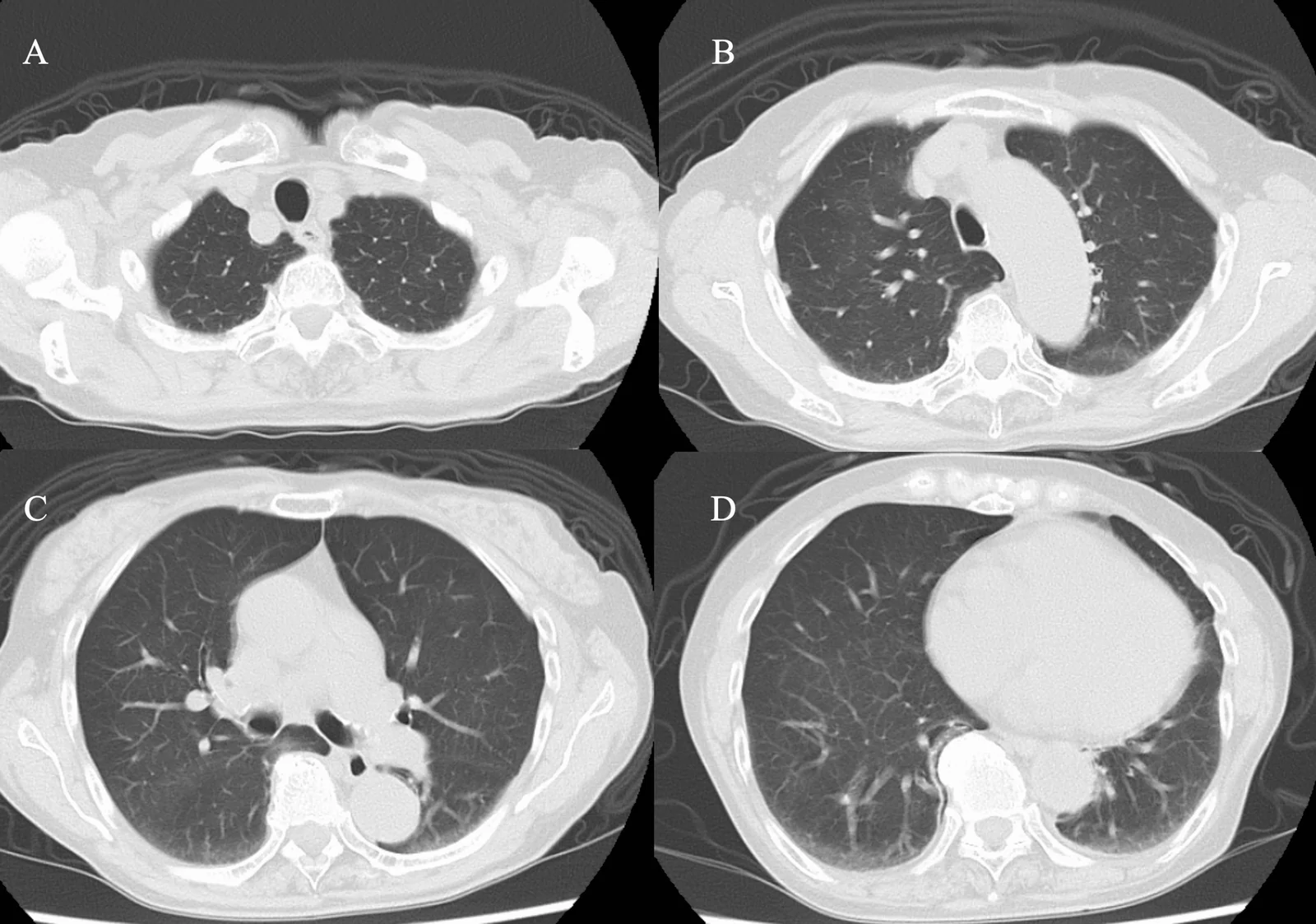
Chest CT findings CT showed no remarkable findings suggestive of pulmonary cryptococcosis in the lungs. (A) Apex. (B) Upper field. (C) Middle field. (D) Lower field CT: computed tomography

Computed tomography also showed an old cerebral infarction in the left thalamus and no abnormalities in the lungs and the abdomen (Figure 2).

Blood tests showed a mildly elevated C-reactive protein level of 0.17 mg/dL (reference range: 0-0.14 mg/dL) and a white blood cell count of 3,000/μL (reference range: 33-86 × 10^2^/μL) (Table 1).

**Table 1:** Blood tests AST: aspartate aminotransferase; ALT: alanine aminotransferase; LDH: lactate dehydrogenase; ALP: alkaline phosphatase; γ-GTP: γ-glutamyl transpeptidase; Ch-E: cholinesterase; CK: creatine kinase; CRP: C-reactive protein; WBC: white blood cell; RBC: red blood cell; Hb: hemoglobin; Ht: hematocrit

Test	Reference range	Result
Total bilirubin	0.4-1.5 mg/dL	1.16 mg/dL
AST	13-30 U/L	19 U/L
ALT	7-23 U/L	16 U/L
LDH	124-222 U/L	234 U/L
ALP	38-113 U/L	109 U/L
γ-GTP	9-32 U/L	21 U/L
Ch-E	201-421 U/L	235 U/L
CK	41-153 U/L	18 U/L
Amylase	33-132 U/L	111 U/L
Total protein	6.6-8.1 g/dL	6.5 g/dL
Albumin	4.1-5.1 g/dL	3.7 g/dL
CRP	0-0.14 mg/dL	0.17 mg/dL
WBC	33-86 × 10^2^/μL	30 × 10^2^/μL
RBC	386-492 × 10^4^/μL	434 × 10^4^/μL
Hb	11.6-14.8 g/dL	12.4 g/dL
Ht	35.1-44.4%	37.1%
Platelet	15.8-34.8 × 10^4^/μL	12.9 × 10^4^/μL

**Figure 3:**
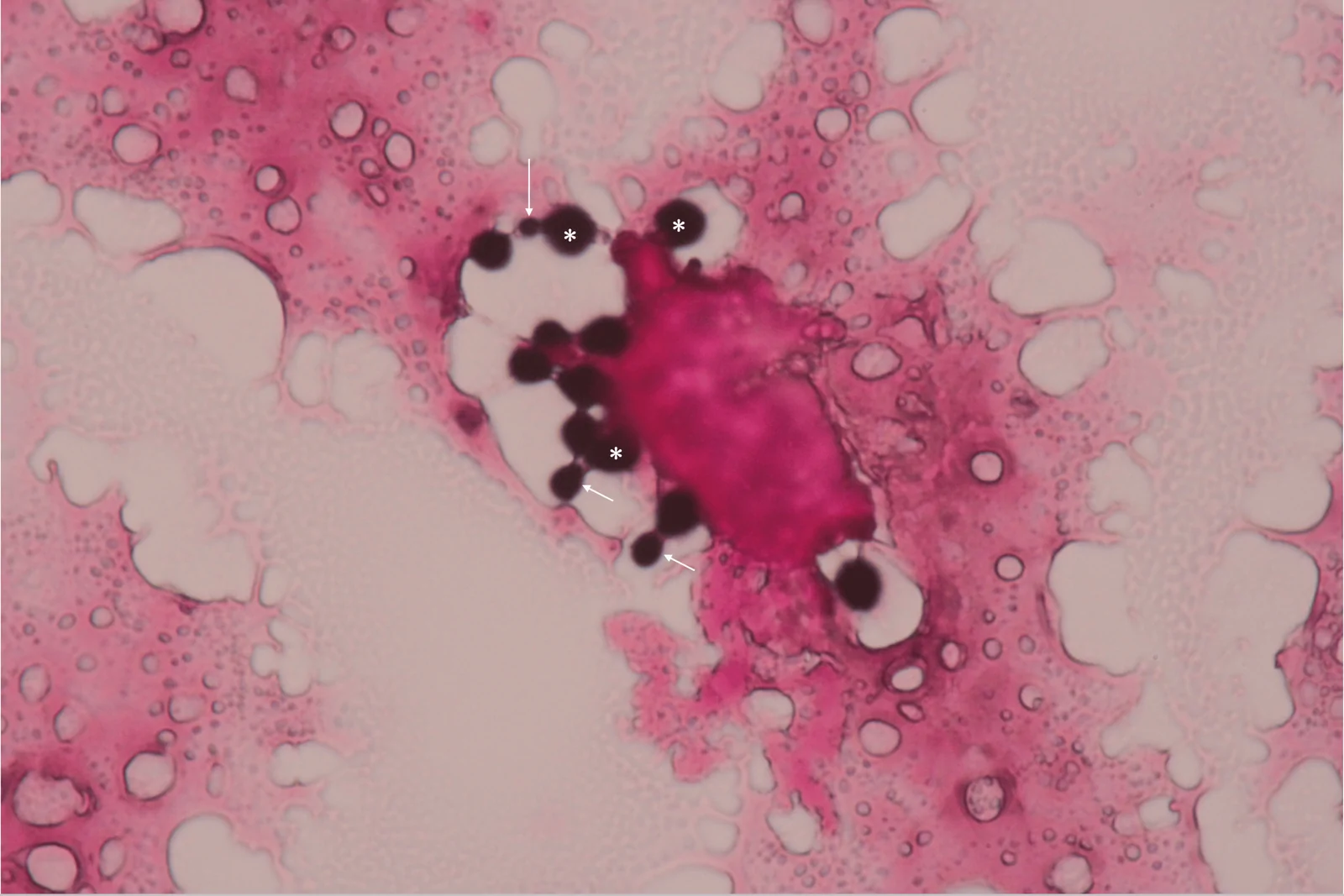
Gram stain findings of the blood culture Gram stain of the blood culture showed multiple Gram-positive fungi (asterisks) with daughter cell budding (arrows) and unclear capsules (×1,000)

Negative influenza A/B and COVID-19 antigen test results, performed to investigate the cause of fever, led us to perform venous blood culture. We hospitalized the patient to receive palliative treatment for various symptoms. After admission, the patient experienced recurrent fever and nausea but did not get symptom relief from nausea even with the administration of antiemetic agents such as metoclopramide. On the fourth day after hospitalization, venous blood culture revealed *C. neoformans* infection (Figure 3).

**Figure 4:**
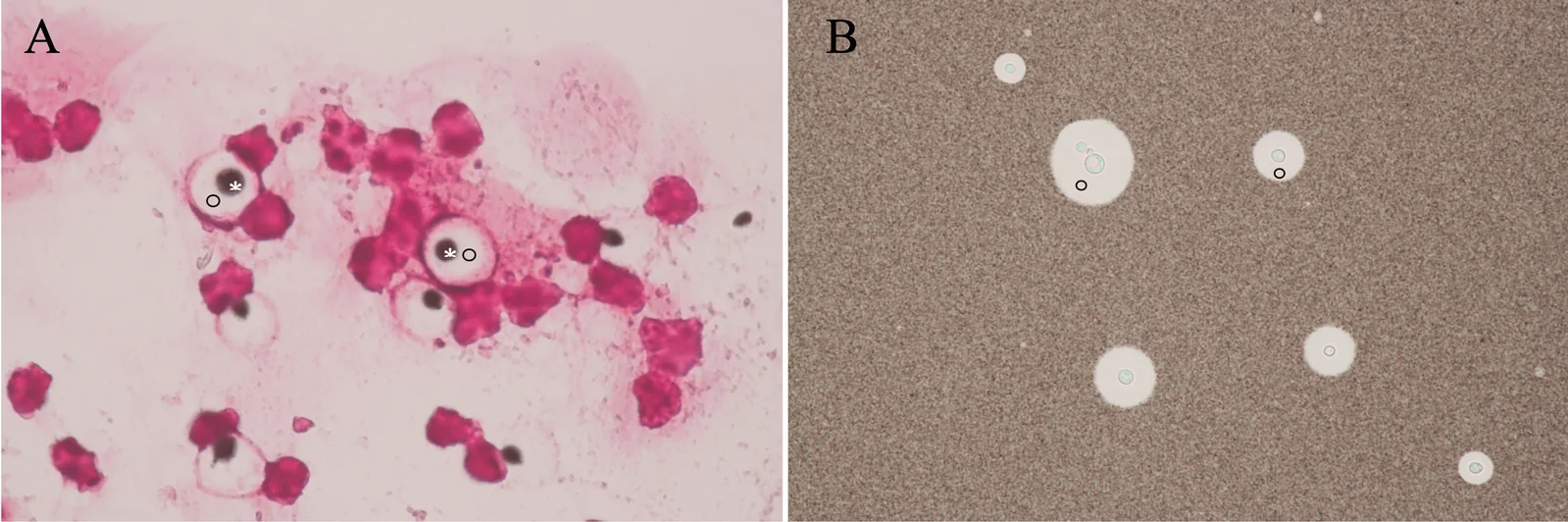
Gram and India stain findings of the cerebrospinal fluid (A) Gram stain of the cerebrospinal fluid also showed Gram-positive fungi (asterisks) with thick capsules (open circles) (×1,000). (B) India ink stain of the cerebrospinal fluid more clearly showed the presence of capsules (open circles) around the fungi (×1,000)

Given the high suspicion of cryptococcal meningitis, a lumbar puncture was performed, which confirmed the presence of *C. neoformans* in the cerebrospinal fluid as well (Figure 4).

Genetic analysis further revealed gene amplification of *Cryptococcus gattii* (Table 2).

**Table 2:** Genetic testing CMV: cytomegalovirus; EV: enterovirus; HSV-1: herpes simplex virus type 1; HSV-2: herpes simplex virus type 2; HHV-6: human herpes virus type 6; HPeV: human parechovirus; VZV: varicella-zoster virus

Items	Results
Escherichia coli	Negative
Haemophilus influenzae	Negative
Listeria monocytogenes	Negative
Neisseria meningitidis	Negative
Streptococcus agalactiae	Negative
Streptococcus pneumoniae	Negative
*Cryptococcus neoformans*/*Cryptococcus gattii*	Positive
CMV	Negative
EV	Negative
HSV-1	Negative
HSV-2	Negative
HHV-6	Positive
HPeV	Negative
VZV	Negative

We, therefore, asked the family members about pigeon keeping after the results of the venous blood culture and cerebrospinal fluid analysis became available. They stated that although they did not have any pigeons, they frequently saw pigeons visiting their balcony and had sometimes cleaned up pigeon droppings. The patient, therefore, began to receive amphotericin B (3.75 mg/kg) and 5-fluorocytosine (25 mg/kg) four times daily. Follow-up cerebrospinal fluid analysis failed to detect fungi by culture, but showed continued gene amplification of *C. neoformans*/*C. gattii* on the 16th day after the initiation of antifungal therapy, followed by neither fungal detection nor its gene amplification on the 25th day. The patient showed no symptom deterioration after the initiation of antifungal therapy, finished it after four weeks, and transferred to another hospital in two months.

## Discussion

This patient developed cryptococcal meningitis without pulmonary cryptococcosis, leading to a difficult diagnosis of cryptococcal meningitis due mainly to the lack of typical meningitis symptoms. Many clinicians know that cryptococcal meningitis can occur without pulmonary cryptococcosis [6,7]. It is also well known that the entry of *C. neoformans* into the oral cavity is the initial step of cryptococcus infection. Few clinicians, however, know the mechanism by which cryptococcal meningitis develops without pulmonary cryptococcosis.

We hypothesize two mechanisms for the development of this specific disorder status. One possible mechanism is that *C. neoformans* reaches the alveoli via the respiratory tract but does not form pulmonary infectious lesions, followed by hematogenous translocation of *C. neoformans* to the meninges through the pulmonary veins into the systemic circulation. This mechanism, however, requires *C. neoformans* to reach the meninges selectively via the external carotid artery [8]. In other words, the absence of cerebral cryptococcosis indicates that there was little or no influx of *C. neoformans* into the internal carotid artery. Taking into account the abundant blood flow of the internal carotid artery compared with that of the external carotid artery, we can conclude that the onset of cryptococcal meningitis without pulmonary cryptococcosis through this mechanism is implausible.

Another possible mechanism involves the direct spread of *C. neoformans* from the oral cavity to the meninges without passing through the lungs. It is well known that the vertebral venous plexus of Batson forms the network of the drainage veins in and around the vertebral bodies, runs along the vertebral column extending from the pelvis to the skull base, is a network of veins lacking internal valve structures, and directly connects the truncal venous system with the intracranial and intraspinal venous system [9]. *C. neoformans*, therefore, reaches the mucosa in the oral cavity, invades its mucosal cells, subsequently proliferates in them, and is released into the extracellular space, followed by translocation to the meninges through Batson's venous plexus. This mechanism well explains cryptococcal meningitis without pulmonary cryptococcosis. This mechanisms naturally often make cryptococcal meningitis not cranial dominant, but spinal dominant, often leading to the lack of typical meningitis symptoms, as in this case.

Breast cancer patients can develop meningeal carcinomatosis, although extremely rarely, without pulmonary and bone metastasis. Some studies have reported that the vertebral venous plexus of Batson should play an important role in the development of carcinomatous meningitis without pulmonary and bone metastasis [10]. However, the incidence of carcinomatous meningitis itself is very low, leading to no systematic analysis of such specific conditions to date. In addition, it is well known that the biology of spinal-type carcinomatous meningitis is somewhat better than that of the cranial type. We believe that a similar trend exists in cryptococcal meningitis, which well explains the lack of typical meningitis symptoms at its earlier phase in this case [11].

We naturally think the pathogenetic mechanisms of the cryptococcal meningitis described in this case report are mere speculation. General practitioners and emergency physicians, however, should always keep in mind the possibility of cryptococcal meningitis caused by this mechanism for earlier diagnosis and better and sooner treatment.

## Conclusions

We experienced a cryptococcal meningitis without pulmonary cryptococcosis, which presented no typical meningitis symptoms. No studies have reported the pathogenetic mechanisms why cryptococcal meningitis develops without the onset of pulmonary cryptococcosis to date. It seems logical that Batson's venous plexus contributes to *C. neoformans* infection to the meninges, like in carcinomatous meningitis. General practitioners and emergency physicians should further note that cryptococcal meningitis caused by this mechanism may present with less typical symptoms of meningitis.
